# Targeting NLRP3 signaling reduces myocarditis-induced arrhythmogenesis and cardiac remodeling

**DOI:** 10.1186/s12929-024-01032-7

**Published:** 2024-04-22

**Authors:** Chye-Gen Chin, Yao-Chang Chen, Fong-Jhih Lin, Yung-Kuo Lin, Yen-Yu Lu, Tzu-Yu Cheng, Shih-Ann Chen, Yi-Jen Chen

**Affiliations:** 1 Graduate Institute of Clinical Medicine, College of Medicine, Taipei Medical University, 250 Wu-Hsing Street, Taipei, 11031 Taiwan; 2 Division of Cardiovascular Medicine, Department of Internal Medicine, Wan Fang Hospital, Taipei Medical University, Taipei, Taiwan; 3 Department of Biomedical Engineering, National Defense Medical Center, Taipei, Taiwan; 4 Division of Cardiology, Department of Internal Medicine, School of Medicine, College of Medicine, Taipei Medical University, Taipei, Taiwan; 5 Division of Cardiology, Department of Internal Medicine, Sijhih Cathay General Hospital, New Taipei City, Taiwan; 6 Division of Cardiovascular Surgery, Wan Fang Hospital, Taipei Medical University, Taipei, Taiwan; 7 Heart Rhythm Center and Division of Cardiology, Department of Medicine, Taipei Veterans General Hospital, Taipei, Taiwan; 8 Division of Cardiology, Taichung Veterans General Hospital, Taichung, Taiwan; 9 Cardiovascular Research Center, Wan Fang Hospital, Taipei Medical University, Taipei, Taiwan

**Keywords:** Myocarditis, NLRP3, Ventricular tachycardia, Right ventricular outflow tract

## Abstract

**Background:**

Myocarditis substantially increases the risk of ventricular arrhythmia. Approximately 30% of all ventricular arrhythmia cases in patients with myocarditis originate from the right ventricular outflow tract (RVOT). However, the role of NLRP3 signaling in RVOT arrhythmogenesis remains unclear.

**Methods:**

Rats with myosin peptide–induced myocarditis (experimental group) were treated with an NLRP3 inhibitor (MCC950; 10 mg/kg, daily for 14 days) or left untreated. Then, they were subjected to electrocardiography and echocardiography. Ventricular tissue samples were collected from each rat’s RVOT, right ventricular apex (RVA), and left ventricle (LV) and examined through conventional microelectrode and histopathologic analyses. In addition, whole-cell patch-clamp recording, confocal fluorescence microscopy, and Western blotting were performed to evaluate ionic currents, intracellular Ca^2+^ transients, and Ca^2+^-modulated protein expression in individual myocytes isolated from the RVOTs.

**Results:**

The LV ejection fraction was lower and premature ventricular contraction frequency was higher in the experimental group than in the control group (rats not exposed to myosin peptide). Myocarditis increased the infiltration of inflammatory cells into cardiac tissue and upregulated the expression of NLRP3; these observations were more prominent in the RVOT and RVA than in the LV. Furthermore, experimental rats treated with MCC950 (treatment group) improved their LV ejection fraction and reduced the frequency of premature ventricular contraction. Histopathological analysis revealed higher incidence of abnormal automaticity and pacing-induced ventricular tachycardia in the RVOTs of the experimental group than in those of the control and treatment groups. However, the incidences of these conditions in the RVA and LV were similar across the groups. The RVOT myocytes of the experimental group exhibited lower Ca^2+^ levels in the sarcoplasmic reticulum, smaller intracellular Ca^2+^ transients, lower L-type Ca^2+^ currents, larger late Na^+^ currents_,_ larger Na^+^–Ca^2+^ exchanger currents, higher reactive oxygen species levels, and higher Ca^2+^/calmodulin-dependent protein kinase II levels than did those of the control and treatment groups.

**Conclusion:**

Myocarditis may increase the rate of RVOT arrhythmogenesis, possibly through electrical and structural remodeling. These changes may be mitigated by inhibiting NLRP3 signaling.

## Background

Myocarditis is a nonischemic cardiomyopathy involving the infiltration of cardiac tissue by inflammatory cells and has infectious, noninfectious, autoimmune, and miscellaneous etiologies. This condition often remains undiagnosed; however, it is detected during approximately 9% of all routine postmortem examinations [1–3]. Myocarditis has heterogeneous clinical manifestations, ranging from nonspecific symptoms to rapidly declining cardiac function and life-threatening arrhythmia [2]. The prevalence of myocarditis is 9%–40% in patients with idiopathic cardiomyopathy [4, 5] and approximately 50% in individuals with ventricular arrhythmia (VA) [6]. In a cohort study, approximately 51% of all patients experiencing frequent premature ventricular contractions (PVCs) had underlying myocardial inflammation [7]. A considerable proportion (30%) of all ventricular arrhythmia cases in patients with myocarditis originate from the right ventricular outflow tract (RVOT) [8]. Evidence suggests an association between COVID-19 and myocarditis [9, 10]: an increased incidence of myocarditis has been discovered in patients with COVID-19, particularly those with severe or critical disease [11]. This observation has drawn attention to the pathogenesis, clinical presentation, and management of COVID-19-associated myocarditis. Myocytes constituting the RVOT exhibit distinct electrophysiological characteristics associated with a high propensity for arrhythmogenesis [12, 13]. However, whether myocarditis promotes RVOT arrhythmogenesis, and thus increases the risk of VA, remains unclear. Moreover, little is known regarding the mechanisms underlying myocarditis-induced VA.

Myocarditis, characterized by inflammation of the cardiac muscle, can trigger a cascade of immune responses that markedly affect the heart’s function and structure. Central to this immune response is activation of the nucleotide oligomerization domain-like receptor family protein 3 (NLRP3) inflammasome; this phenomenon has been increasingly recognized as a key mediator of the myocardial inflammatory response [14]. NLRP3 is an intracellular signaling molecule that senses various factors related to the environment, host, and pathogens [15]. Inflammasomes recruit procaspase-1 through adaptor molecule apoptosis–associated speck-like protein containing a C-terminal caspase recruitment domain and then cleave the cytokine precursors prointerleukin-1β and prointerleukin-18 into mature interleukin (IL)-1β and IL-18, respectively. Thus, inflammasomes are critical components of the inflammatory process. Activation of the NLRP3 inflammasome plays a vital role in the pathogenesis of cardiac arrhythmia. Activation of the NLRP3 inflammasome is upregulated in the atria of patients with atrial fibrillation, and inhibition of NLRP3 signaling has been demonstrated to mitigate atrial fibrillation in animal models [16]. Myocarditis may promote arrhythmogenesis in the RVOT, thereby increasing the risk of VA. Research on this topic is ongoing, with many aspects remaining to be clarified. Similarly, the precise role of the NLRP3 inflammasome in the pathogenesis of VA is yet to be elucidated. We hypothesize that activation of the NLRP3 inflammasome and the subsequently released proinflammatory cytokines mediate the pathogenesis of VA by altering the function of ion channels. Alterations to ion channel function may alter cardiac excitability, thereby facilitating the development of arrhythmia. Although inflammation has been implicated in myocarditis, the specific roles of various inflammatory processes and the NLRP3 inflammasome in the pathogenesis of myocarditis and this condition’s progression to arrhythmogenesis, particularly in the RVOT, remain underexplored. This knowledge gap underscores the need for further studies to clarify how myocarditis increases the risk of VA through inflammasome activation and its effects on cardiac electrophysiology. Accordingly, in this study, we investigated how myocarditis leads to ventricular arrhythmogenesis both in vitro and in vivo and explored the relevant therapeutic benefits of targeting NLRP3 signaling.

## Methods

### Establishment of a rat model of acute myocarditis

**Fig. 1:**
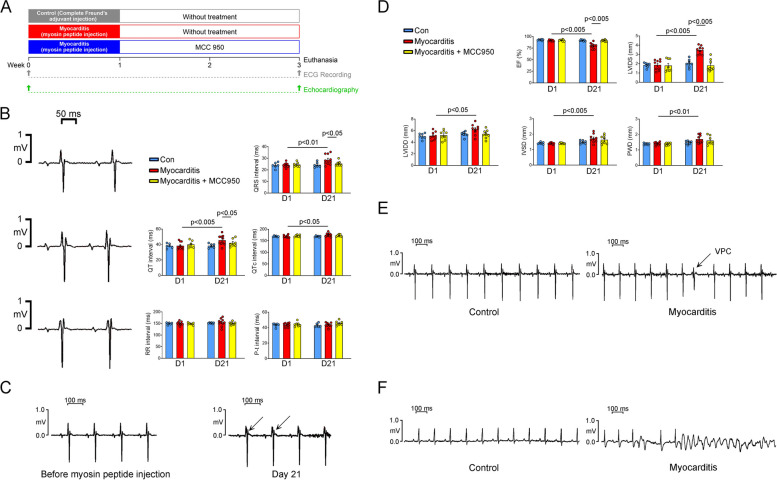
Study design and in vivo electrophysiology. **A** Experimental protocol. **B** Echocardiographic parameters of the control (healthy rats; *n* = 7), experimental (rats with myocarditis; *n* = 9), and treatment (MCC950-treated rats with myocarditis; *n* = 8) groups. **C** ECG readings before and after myosin peptide injection; rats with myocarditis had abnormal ECG morphologies with QRS fragmentation. **D** Representative electrocardiographic tracings and average data of the control (*n* = 7), experimental (*n* = 7), and treatment (*n* = 7) groups on days 1 (baseline) and 21 (after treatment). **E** ECG tracing revealing spontaneous premature ventricular contractions in a rat with myocarditis. **F** ECG tracing indicating caffeine (120 mg/kg)-induced ventricular tachycardia in a rat with myocarditis. ECG, electrocardiography

The study protocol was reviewed and approved by the Institutional Animal Care and Use Committee of Taipei Medical University (animal use permission number: LAC-2020–0416). Male Lewis rats (age, 8 weeks) were purchased from BioLASCO (Taiwan). The rats were divided into experimental and control groups. On days 1 and 7, the experimental group received tail-vein injections of myosin peptide [17] (100 μg of peptide dissolved in 0.2 mL of complete Freund’s adjuvant [CFA; widely used to induce autoimmune disease in animal models]), whereas the control group received tail-vein injections of 0.2 mL of CFA. Some rats in the experimental group were treated with an NLRP3 inhibitor (MCC950; Apexbio; 10 mg/kg, daily from days 8 to 21; treatment group) [18, 19], whereas others were left untreated to enable comparison. All rats were euthanized on day 21 by using an overdose of isoflurane (5% in O_2_). A flowchart depicting the experimental process is presented in Fig. 1A.

### Ambulatory electrocardiography telemetry and echocardiography

Telemetry transmitters (HD-S21; Data Sciences International, St. Paul, MN, USA) were implanted in the rats by following the manufacturer’s protocol. The transmitters were placed intraperitoneally, and a lead II configuration was adopted for electrocardiography (ECG). ECG data were collected and processed using LabChart (version 8; Data Sciences International, Dunedin, New Zealand). Transthoracic echocardiography was performed using a Vivid I ultrasound cardiovascular system (GE Healthcare, Haifa, Israel) on days 1 and 21 [20].

### Electropharmacological analyses

For sectioning, the endocardial side of the rat hearts was placed facing upward. The RVOT was sliced approximately 1 to 2 mm below the pulmonary valve. The right ventricular apex (RVA) and left ventricle (LV) were sectioned in the region near the ventricular apex, proximal to the cavity. The RVOT, RVA, and LV tissue preparations (~ 1 cm × 1.5 cm) were incubated at 37 °C in Tyrode’s solution containing 137 mM NaCl, 4 mM KCl, 15 mM NaHCO_3_, 0.5 mM NaH_2_PO_4_, 0.5 mM MgCl_2_, 2.7 mM CaCl_2_, and 11 mM dextrose. One end of each tissue preparation was fixed in a tissue bath, and the other end was connected through silk thread to the Grass FT03C force displacement transducer (Grass Instrument, Quincy, MA, USA). The tissue was perfused with Tyrode’s solution at a constant rate of 3 mL/min; the solution was saturated with a gas mixture comprising 97% O_2_ and 3% CO_2_. Before electrophysiological analysis was performed, the tissue preparations were allowed to equilibrate for 1 h. The transmembrane action potentials (APs) of the isolated preparations were recorded using machine-pulled glass capillary microelectrodes filled with 3 M KCl. These microelectrodes were connected to an electrometer (model FD223; World Precision Instruments) under 150 mg of tension, as described previously [21]. The AP and contraction force were simultaneously recorded using the Tektronix TDS2000C digital storage oscilloscope and the Gould ES1000 recorder (OH, USA). Signals from the microelectrode leads were digitally recorded with 16-bit accuracy at a frequency of 125 kHz.

The AP duration (APD) was measured at 20% (APD_20_), 50% (APD_50_), and 90% (APD_90_) of full repolarization relative to the peak amplitude of the AP. The contractility amplitude was measured from the stable baseline to the peak of a contraction. Electrical stimulation was performed using a 1-ms pulse originating from a Grass S88 stimulator (Grass Instrument) through a Grass SIU5B stimulus isolation unit. Electrical stimuli were delivered at a frequency of 2 Hz for 20 consecutive beats within 1 s, both with and without the administration of rapamycin (100 nM; used to dissociate the ryanodine receptor [RyR]– FK506-binding protein 12.6 complex).

### Immunohistological analyses

The hearts were fixed with 10% buffered formalin, and 5-μm-thick paraffin-embedded sections of LV, RVA, and RVOT tissues were prepared. Each rat and its isolated heart were weighed, and the corresponding heart weight/body weight ratio was calculated. Subsequently, the heart sections were stained with hematoxylin–eosin. Furthermore, the tissue sections were immunostained with antibodies against CD45 (catalog no. ab10558) to examine the distribution of white blood cells in cardiac tissue for assessing inflammatory response and immune cell infiltration. The sections were also immunostained with antibodies against NLRP3 (catalog no. 19771–1-AP; dilution, 1:500; negative control). The levels of CD45 and NLRP3 expression were measured using Image-Pro Plus (version 6.0; Media Cybernetics, Rockville, MD, USA).

### Isolation of individual myocytes from the RVOT

After the rats were euthanized, the rat hearts were promptly excised and dissected at room temperature in normal Tyrode’s solution containing 140 mM NaCl, 5.4 mM KCl, 1.8 mM CaCl_2_, 1 mM MgCl_2_, 10 mM glucose, and 10 mM HEPES (pH 7.4); all ingredients were purchased from Sigma-Aldrich. This solution was equilibrated using 100% O_2_, and a pH of approximately 7.4 was maintained. Individual cardiomyocytes were enzymatically dissociated from the RVOT. The cells were allowed to stabilize in a tissue bath for a minimum of 30 min before the experiments were conducted.

### Measurement of intracellular Ca^2+^ transients

Ventricular myocytes isolated from the RVOT were loaded with a fluorescent Ca^2+^ indicator (fluo-3/AM; concentration, 10 μM in Tyrode’s solution). The loading process was performed at room temperature and lasted for 30 min [22]. The fluorescent indicator was excited at 488 nm by using an argon ion laser, and fluorescence signals were recorded at wavelengths of ≥ 15 nm. The cells were repeatedly scanned at 2-ms intervals for a total duration of 6 s. Fluorescence imaging was performed using a laser scanning confocal microscope (TCS SP5; Leica Microsystems, Wetzlar, Germany). To account for variation in the dye concentration, the fluorescent signals were corrected by normalizing the measured fluorescence (*F*) against baseline fluorescence (*F*_0_), which yielded a reliable estimate of changes in intracellular Ca^2+^ ([Ca^2+^]_i_) transients relative to baseline values ([*F* − *F*_0_]/*F*_0_) and ensuring minimal variation in fluorescence intensity due to differences in dye volumes. [Ca^2+^]_i_ transients and peak systolic and diastolic [Ca^2+^]_i_ levels were measured during 1-Hz field stimulation with 10-ms square-wave pulses at twice the threshold strength. After steady-state [Ca^2+^]_i_ transients had been achieved through repeated pulses from − 40 to 0 mV (1 Hz for 5 s), the level of Ca^2+^ in the sarcoplasmic reticulum (SR) was estimated. The release of Ca^2+^ from the SR was induced by first treating the cells with 20 mM caffeine for 0.5 s while maintaining the membrane potential at − 40 mV and then integrating Na^+^–Ca^2+^ exchanger (NCX) current [23]. The time integral of the NCX current was converted into amoles of Ca^2+^ released from the SR [24]. The leakage of Ca^2+^ from the SR was measured as described previously [22]. In brief, after steady-state [Ca^2+^]_i_ transients had been achieved through repeated pulses (at 1 Hz for 5 s), the Na^+^-free and Ca^2+^-free solution was supplemented with tetracaine (1 mM) to measure the tetracaine-induced reduction in [Ca^2+^]_i_ level, which represented the leakage of Ca^2+^ from the SR.

### Whole-cell patch-clamp recordings of individual myocytes

RVOT myocytes isolated from the rats were enzymatically dissociated, as described previously [25]. Individual myocytes were subjected to whole-cell perforated patch-clamp recording, which was performed using the Axopatch 1D amplifier (Axon Instruments, Foster City, CA, USA) at 35 °C ± 1 °C [22]. Ionic currents were recorded at a similar time interval (approximately 3–5 min) after amphotericin B (300 μg/mL)-induced cell rupture or perforation (for the L-type Ca^2+^ current [*I*_Ca-L_]) to maintain the activity of ion channels. Ionic currents were measured in the voltage-clamp mode.

To measure NCX current, micropipettes were filled with solution containing 20 mM NaCl, 110 mM CsCl, 0.4 mM MgCl_2_, 1.75 mM CaCl_2_, 20 mM tetraethylammonium chloride, 5 mM 1,2-bis(2-aminophenoxy)ethane-N,N,N′, N′-tetra-acetic acid, 5 mM glucose, 5 mM MgATP, and 10 mM HEPES; the solution was titrated to pH 7.25 with CsOH. In the electrophysiological experiments, the access resistance was approximately 1.4 ± 0.1 MΩ. To measure *I*_Ca-L_, micropipettes were filled with solution containing 130 mM CsCl, 1 mM MgCl_2_, 5 mM MgATP, 10 mM egtazic acid, 0.1 mM NaGTP, 5 mM Na_2_ phosphocreatine, and 10 mM HEPES; the solution was titrated to pH 7.2 with CsOH. To measure late Na^+^ current (*I*_Na‐Late_), micropipettes were filled with solution containing 10 mM NaCl, 130 mM CsCl, 5 mM egtazic acid, 5 mM glucose, 5 mM MgATP 5, and 5 mM HEPES; the solution was titrated to pH 7.3 with NaOH.

*I*_Na‐Late_ was recorded at room temperature by using external solution containing 130 mM NaCl, 5 mM CsCl, 1 mM MgCl_2_, 1 mM CaCl_2_, 10 mM glucose, and 10 mM HEPES; the solution was titrated to pH 7.4 with NaOH. The current was recorded using a step–ramp protocol (− 100 mV stepped to + 20 mV for 100 ms before ramping it back to − 100 mV over a 100-ms period). An equilibration period for dialysis was included to ensure the correct clamping of cell currents. *I*_Na‐Late_ was measured in terms of the tetrodotoxin (30 µM)-sensitive portions of the current traces obtained as the current was ramped back to − 100 mV, as described previously [26].

*I*_Ca-L_ was measured as an inward current during depolarization from a holding potential of − 50 mV to test potentials ranging from − 40 to + 60 mV in 10‐mV steps over a 300-ms period at a frequency of 0.1 Hz. For this, we used a perforated patch-clamp with amphotericin B (300 μg/mL). NaCl and KCl present in the external solution were replaced by tetraethylammonium chloride and CsCl, respectively. The peak current amplitude was selected to represent *I*_Ca-L_.

The NCX current was elicited using test pulses ranging from − 100 to + 100 mV from a holding potential of − 40 mV over a 300-ms period at a frequency of 0.1 Hz. Notably, the NCX current was estimated by subtracting the currents sensitive to 10 mM Ni^2+^ under control conditions from those recorded in the presence of Ni^2+^. This approach helped measure the exchange of Na^+^ and Ca^2+^ ions in terms of the NCX current. The external solution comprised 140 mM NaCl, 2 mM CaCl_2_, 1 mM MgCl_2_, 10 mM glucose (pH 7.4), 10 μM strophanthidin, 10 μM nitrendipine, 100 μM niflumic acid, and 5 mM HEPES.

### Measurement of intracellular reactive oxygen species and Na^+^ level

Cross-sectional areas of the isolated RVOT myocytes were visualized using a confocal laser scanning microscope (Zeiss LSM 510; Carl Zeiss). The images were processed using ImageJ [20]. CellROX green (Life Technologies, Grand Island, NY, USA) was used to measure the cytosolic levels of reactive oxygen species (ROS), whereas MitoSOX Red (Life Technologies) was used to measure the mitochondrial levels of ROS. Asante NaTRIUM Green-2 AM (Teflabs, Austin, TX, USA) was employed to measure the cytosolic levels of Na^+^ in RVOT myocytes freshly isolated from the control, experimental, and treatment groups. Microscopic examinations were performed using the aforementioned Zeiss microscope and an inverted microscope (Axiovert 100) with a 63 × 1.25 numerical aperture oil immersion objective, as described previously [27]. CellROX green, MitoSOX Red, and Asante NaTRIUM Green-2 were excited at 488 nm, and fluorescence signals were acquired at a wavelength of ≥ 505 nm by using the confocal system in the XY mode. During the experiment, RVOT myocytes were paced at a frequency of 1 Hz. Fluorescence images were analyzed using Image-Pro Plus (version 6.0) and SigmaPlot (version 12), as described previously [20].

### Western blotting

Changes in the expression of ion channels and Ca^2+^-modulated proteins were investigated through Western blotting. RVOT tissues were homogenized and centrifuged in buffer systems. Equal amounts of total protein were separated through 5% or 8% sodium dodecyl sulfate–polyacrylamide gel electrophoresis. The resultant protein bands were electrophoretically transferred onto polyvinylidene difluoride membranes. For the immunofluorescence-based detection of gap junction proteins, all blots were stained with primary antibodies against sarcoplasmic/endoplasmic reticulum Ca^2+^ ATPase 2a (SERCA2a), Ca^2+^/calmodulin-dependent protein kinase II (CaMKII) phosphorylated at Thr286 (pCaMKII), RyR2, total phospholamban (Thermo Fisher Scientific, Rockford, IL, USA), phosphorylated RyR at Ser2808, phospholamban phosphorylated at Thr17 (Badrilla, UK), catalytic subunit of protein kinase A (BD BioSciences, USA), Cav1.2, NLRP3, nuclear factor (NF)-κB, IL-1β, and glyceraldehyde-3-phosphate dehydrogenase (MBL, Japan); all secondary antibodies were conjugated with horseradish peroxidase. Bound antibodies were detected using an enhanced chemiluminescence detection system and were analyzed using the AlphaEaseFC software. To ensure equal protein loading, all target bands were normalized to a band of glyceraldehyde-3-phosphate dehydrogenase.

### Statistical analysis

All continuous variables are expressed as means ± standard deviations. One-way analysis of variance with a post hoc Tukey test was employed to compare variables between the control, myocarditis, and MCC950-treated myocarditis groups. Differences between nonparametric variables were analyzed using the chi-square test with Fisher’s exact correction. *P* < 0.05 was considered statistically significant.

## Results

### NLRP3 inhibition mitigates myocardial dysfunction and prevents VA in rats with myocarditis

Our echocardiographic analysis of LV function revealed markedly lower LV ejection fractions, greater LV septal wall thickness, and larger left ventricular internal diameters at end diastole and systole in the experimental group than in the control group (Fig. 1D). However, MCC950 treatment significantly mitigated the dysfunction of the LV and the dilation of the left ventricular internal diameters at end diastole and systole (Fig. 1D). In vivo monitoring of the experimental group indicated abnormal ECG morphologies on day 21 (Fig. 1C), characterized by QRS fragmentation—a typical ECG finding for myocarditis. The experimental group also exhibited longer QT intervals, corrected QT intervals (QTc), and QRS durations than did the control and treatment groups (Fig. 1B). Notably, the three groups had similar RR and PR intervals, suggesting that overall heart rate and atrioventricular conduction were not significantly affected by myocarditis or MCC950 treatment. Furthermore, after receiving an intraperitoneal injection of caffeine (120 mg/kg), the experimental group (*n* = 6) exhibited a higher rate of VA incidence than did the control (*n* = 6) and treatment (*n* = 10) groups; the groups were compared in terms of spontaneous PVCs (50% vs. 0% and 0%, respectively; *P* < 0.05 for both) and ventricular tachycardia (VT; 20% vs. 0% [*P* = 0.145] and 0% [*P* = 0.12], respectively; Fig. 1E,F). These findings suggest that caffeine increases the risk of myocarditis-associated arrhythmogenesis in rats.

### Myosin peptide evokes an inflammatory response during early stage myocarditis in rats

**Fig. 2:**
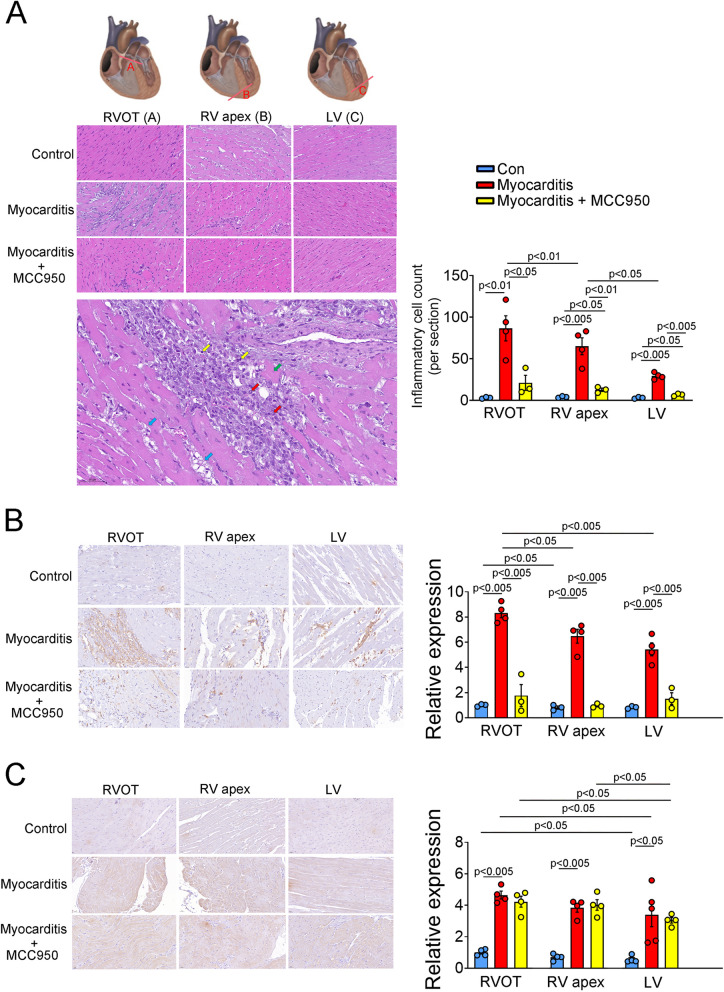
Results of hematoxylin–eosin and immunohistochemical staining performed to measure NLRP3 expression. Representative sections (day 21) of the RVOTs, RVAs, and LVs of the control (healthy rats), experimental (rats with myocarditis), and treatment (MCC950-treated rats with myocarditis) groups after hematoxylin–eosin staining and immunohistochemical staining for CD45 and NLRP3 (magnification, 400 ×). **A** Average counts of inflammatory cells (lymphocytes and macrophages) in the control (*n* = 3), experimental (*n* = 4), and treatment (*n* = 3) groups. Macrophages (red arrow) and lymphocytes (yellow arrow) were observed in addition to interstitial space edema (blue arrow) and cardiomyocyte necrosis (green arrow). **B** Relative expression of CD45 in the RVOTs of the control group was compared with that in different heart regions of the experimental and treatment groups. Average CD45 immunostaining results of the control (*n* = 4), experimental (*n* = 4), and treatment (*n* = 4) groups. **C** Relative expression of NLRP3 in the RVOTs of the control group was compared with that in the experimental and treatment groups. Average NLRP3 immunostaining results of the control (*n* = 4), experimental (*n* = 4), and MCC950-treated (*n* = 4) groups. RVOT, right ventricle outflow tract; RVA, right ventricular apex; LV, left ventricle

Histological analysis revealed the infiltration of immune cells into the cardiac tissue of the experimental group on day 21 (Fig. 2). This infiltration, indicative of myocardial inflammation, was accompanied by localized expansion of the interstitial space due to cardiomyocyte necrosis, a hallmark of the “infarct-like” phenotype associated with myocarditis. Notably, we observed inflammatory cells such as lymphocytes and macrophages within the myocardium. The degree of inflammatory cell infiltration was significantly higher in the experimental group than in the control group; however, this increase was mitigated by MCC950 treatment (Fig. 2A,B). Furthermore, in the experimental group, a more pronounced inflammatory response was observed in the RVOT than in the LV. MCC950 treatment effectively alleviated this inflammatory response. Immunohistochemical staining revealed significantly higher levels of NLRP3 expression in the experimental and MCC950 treatment groups than in the control group. In the experimental group, the level of NLRP3 expression was significantly higher in the RVOT than in the LV (Fig. 2C). This finding suggests variation in NLRP3 expression across cardiac regions in rats with myocarditis.

### AP morphologies in the RVA, RVOT, and LV of the study groups

**Fig. 3:**
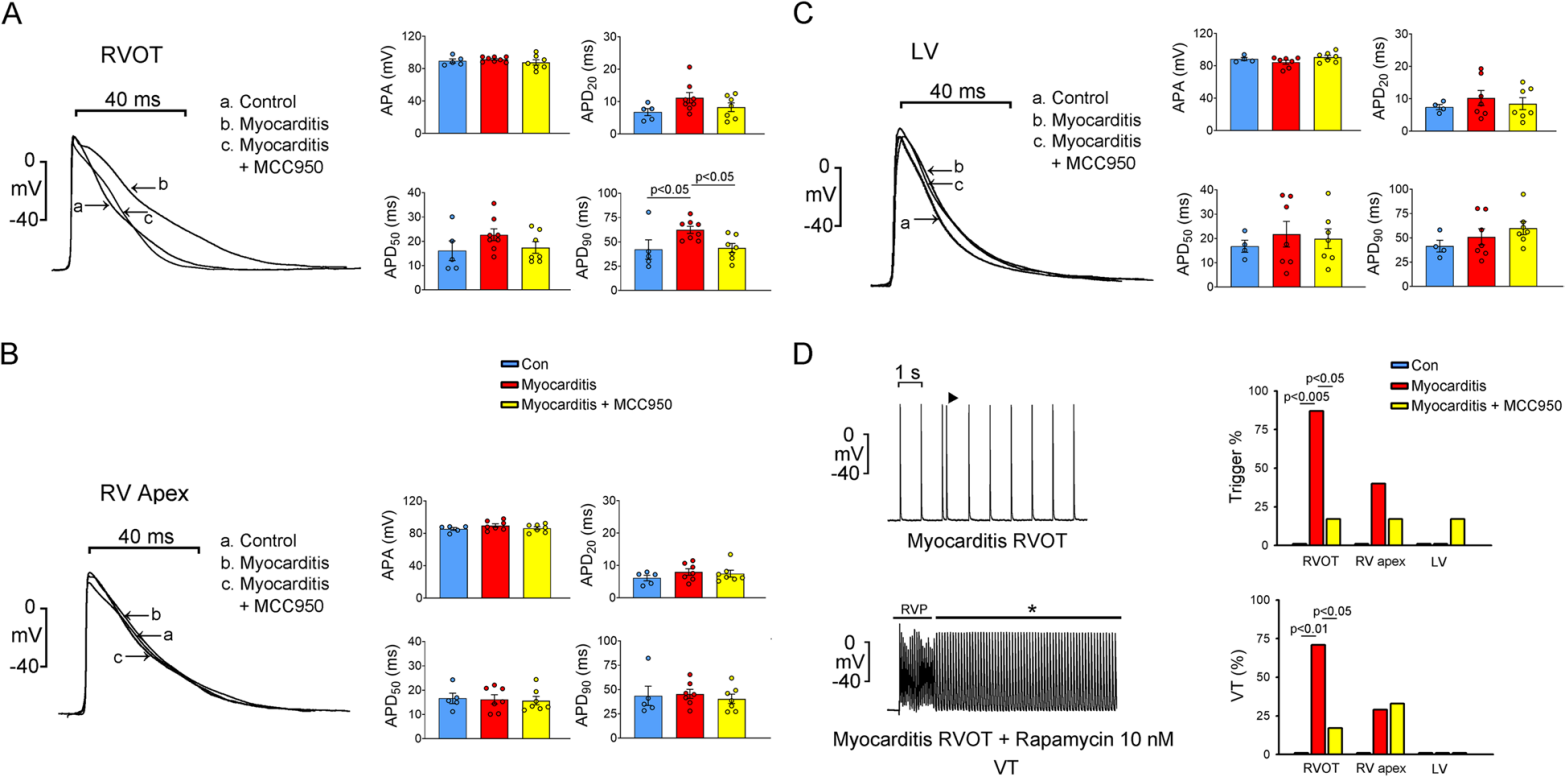
Morphology of AP and incidence of premature ventricular contractions and ventricular tachycardia. **A** Superimposed traces depicting APs in the RVOTs of the control (healthy rats), experimental (rats with myocarditis), and treatment (MCC950-treated rats with myocarditis) groups (*n* = 8). APA and APD at repolarizations of 20%, 50%, and 90% (APD_20_, APD_50_, and APD_90_) are indicated. **B** Superimposed traces depict APs in the RVAs of the control, experimental, and treatment groups (*n* = 8). APA, APD_20_, APD_50_, and APD_90_ are indicated. **C** Superimposed traces depict APs in the LVs of the control, experimental, and treatment groups (*n* = 8). APA, APD_20_, APD_50_, and APD_90_ are indicated. **D** Premature ventricular contractions and ventricular tachycardia in the RVOTs, RVAs, and LVs of the control (*n* = 6), experimental (*n* = 10), and treatment (*n* = 7) groups. AP, action potential; APA, action potential amplitude; APD, action potential duration; RVOT, right ventricle outflow tract; RVA, right ventricular apex; LV, left ventricle

The RVOTs of the experimental group exhibited a longer APD90 than did the RVOTs of the control and treatment groups (Fig. 3). However, similar APD50 and APD20 values were found in the three groups. Moreover, no significant between-group difference was observed in the APD20, APD50, or APD90 corresponding to the RVA or LV. The triggered activity rates were higher in the RVOTs of the experimental group than in the RVOTs of the control and treatment groups. However, similar rates of triggered activity in the RVAs and LVs were observed among the groups (Fig. 3). Rapamycin treatment (100 nM) resulted in a 20-beat pause following 2-Hz field stimulation, leading to the induction of VT in the RVOTs of the experimental group but not those of the control group. However, during pacing and after rapamycin treatment, the rate of VT incidence was lower in the RVOTs of the treatment group than in the RVOTs of the experimental group. Notably, the same pause protocol led to a lower rate of VT incidence in the RVAs than in the RVOTs but no VT incidence in the LVs. Thus, the RVOT may be more susceptible to myocarditis-induced arrhythmia than are other ventricular regions. MCC950 treatment appears to effectively mitigate the myocarditis-induced increase in the risk of arrhythmia (Fig. 3).

### Effects of myocarditis on RVOT electrical activity and intracellular ROS production

**Fig. 4:**
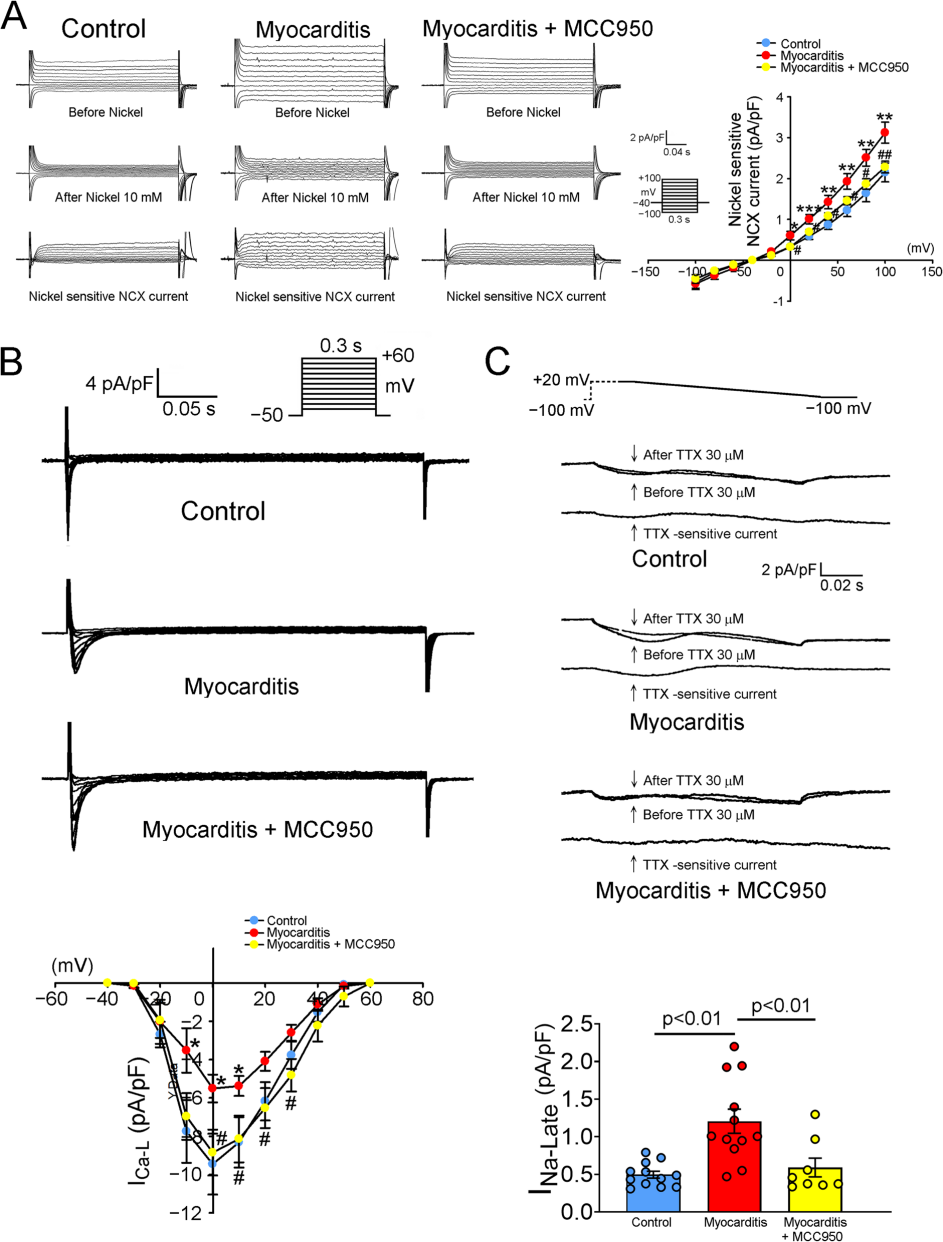
Estimates of *I*_Na-Late_, *I*_Ca-L_, and NCX current in rat RVOTs. **A** Tracings and *I*–*V* relationship of NCX current in the RVOT cardiomyocytes of the control (healthy rats; *n* = 10), experimental (rats with myocarditis; *n* = 10), and treatment (MCC950-treated rats with myocarditis; *n* = 9) groups. **B** Tracings and *I*–*V* relationship of *I*_Ca-L_ in the RVOT cardiomyocytes of the control (*n* = 14), experimental (*n* = 16), and treatment (*n* = 12) groups **C** Current tracings and average data of *I*_Na-Late_ in the RVOT cardiomyocytes of the control (*n* = 12), experimental (*n* = 12), and treatment (*n* = 8) groups. ***I***_**Na-Late**_, late Na^+^ current; *I*_Ca-L_, L-type Ca^2+^ current; NCX, Na^+^–Ca^2+^ exchanger; RVOT, right ventricle outflow tract

**Fig. 5:**
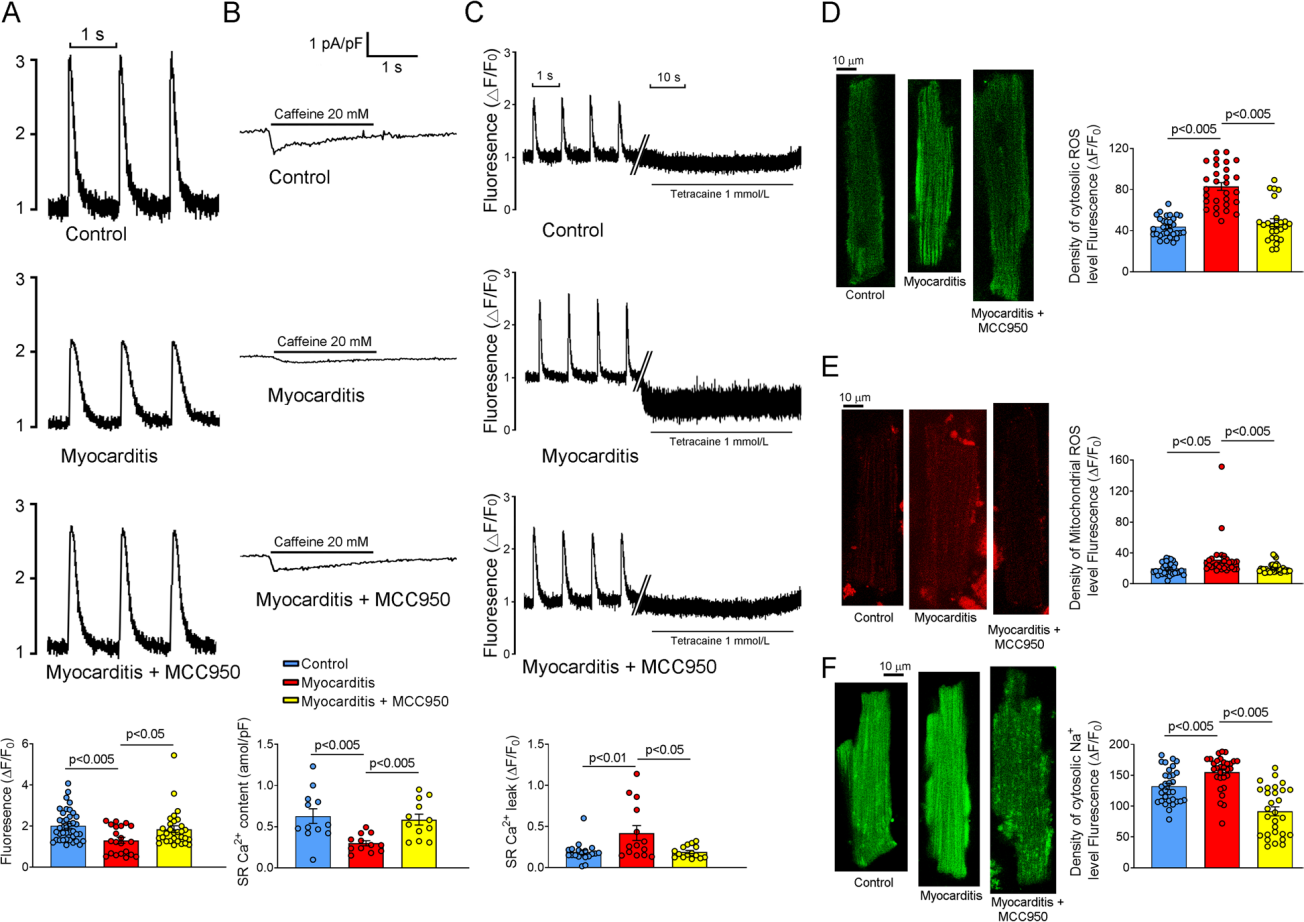
ROS, intracellular Ca^2+^, SR Ca^2+^, Ca^2+^ leak, and cytosolic Na^+^ levels in rat RVOTs. **A** Tracings and average levels of [Ca^2+^]_i_ transients and fractional SR Ca^2+^ release in the RVOT cardiomyocytes of the control (*n* = 30), experimental (rats with myocarditis; *n* = 28), and treatment (MCC950-treated rats with myocarditis; *n* = 30) groups. **B** Tracings and average SR Ca^2+^ levels in the RVOT cardiomyocytes of the control (*n* = 12), experimental (*n* = 12), and treatment (*n* = 12) groups. **C** Tracings and average Ca^2+^ leak levels in the RVOT cardiomyocytes of the control (*n* = 19), experimental (*n* = 14), and treatment (*n* = 15) groups. **D** Average levels of cytosolic ROS in the RVOT cardiomyocytes of the control (*n* = 30), experimental (*n* = 31), and treatment (*n* = 25) groups. **E** Average levels of mitochondrial ROS in the RVOT cardiomyocytes of the control (*n* = 33), experimental (*n* = 30), and treatment (*n* = 33) groups. **F** Average levels of cytosolic Na^+^ in the RVOT cardiomyocytes of the control (*n* = 30), experimental (*n* = 30), and treatment (*n* = 29) groups. ROS, reactive oxygen species; SR, sarcoplasmic reticulum; RVOT, right ventricle outflow tract

In the RVOTs of the experimental group, we observed several electrophysiological alterations. Specifically, the experimental group exhibited considerably larger *I*_Na-Late_, larger reverse-mode NCX current, and smaller *I*_Ca-L_ than did the control group. However, MCC950-treated rats exhibited smaller *I*_Na-Late_, lower NCX activity, and higher *I*_Ca-L_ than did the untreated experimental rats (Fig. 4). These findings suggest that MCC950 treatment can mitigate myocarditis-associated electrophysiological abnormalities in the RVOT of rats. Furthermore, the RVOTs of the experimental group had higher levels of intracellular and mitochondrial ROS than did the RVOTs of the control and treatment groups (Fig. 5 D,E). Therefore, MCC950 treatment mitigated the myocarditis-induced increase in oxidative stress within rat RVOTs. Similarly, the intracellular level of Na^+^ in the RVOTs of the experimental group was higher than that in the RVOTs of the control and treatment groups (Fig. 5F).

### Effects of MCC950 on Ca^2+^ levels in rats with myocarditis

The RVOTs of the experimental group had smaller [Ca^2+^]_i_ transients than did the RVOTs of the control and treatment groups. However, the control and treatment groups exhibited similar [Ca^2+^]_i_ transients and SR Ca^2+^ levels (Fig. 5 A,B). To elucidate the underlying mechanisms, we measured the levels of Ca^2+^ in the SR and found that these levels were lower in the RVOTs of the experimental group than in the RVOTs of the control and treatment groups. The experimental group also exhibited a larger Ca^2+^ leak than did the control and treatment groups (Fig. 5C), suggesting that myocarditis reduces [Ca^2+^]_i_ transients and SR Ca^2+^ levels by increasing Ca^2+^ leakage. These findings indicate that myocarditis results in reduced [Ca^2+^]_i_ transients and SR Ca^2+^ levels in RVOT myocytes. Inhibition of NLRP3 signaling may mitigate the effects of myocarditis on Ca^2+^ handling and restore Ca^2+^ homeostasis.

### Effects of MCC950 on Ca^2+^ regulatory proteins in rats with myocarditis

**Fig. 6:**
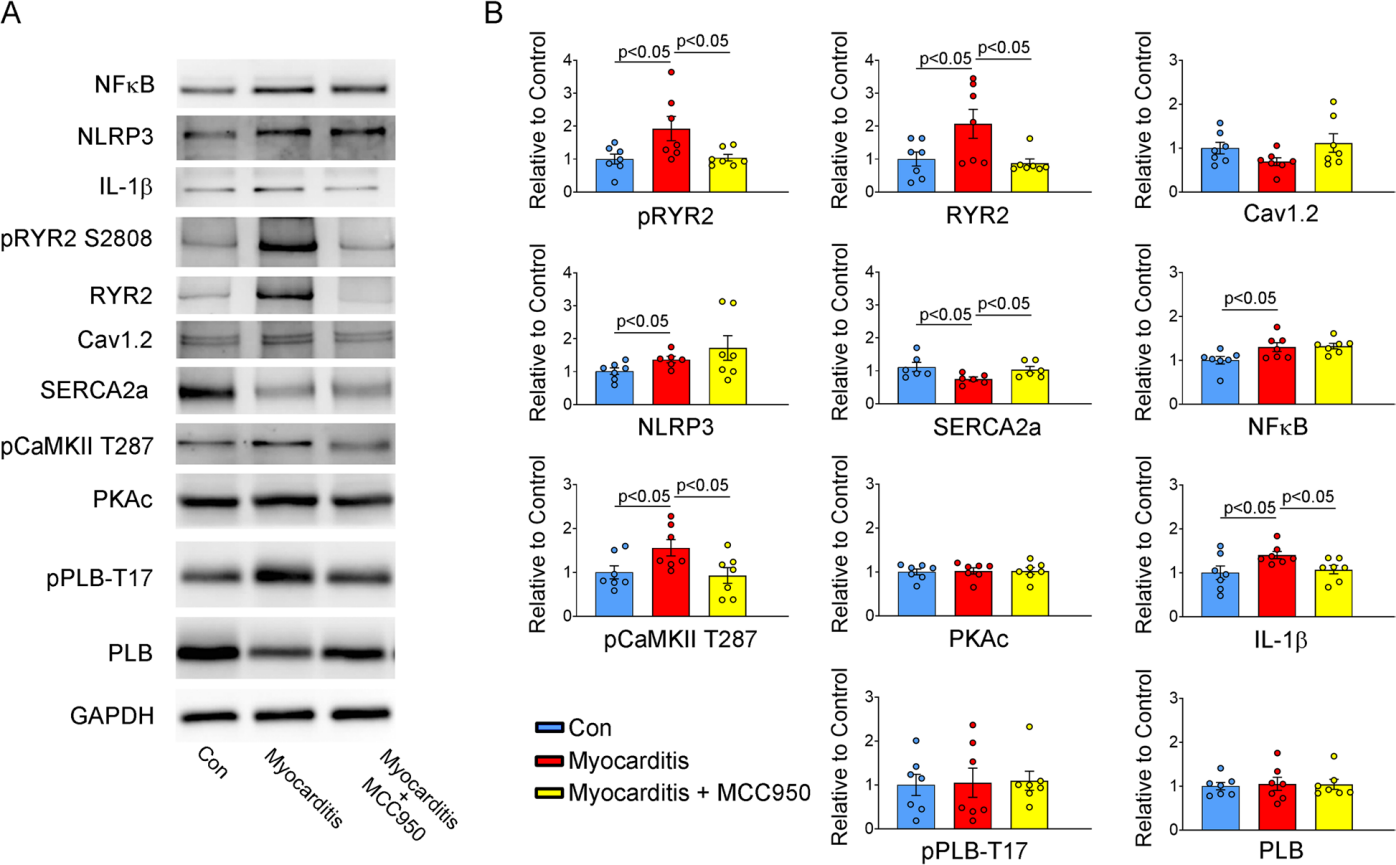
Effects of the NLRP3/CaMKII axis on Ca^2+^ regulatory proteins in rat RVOTs. Representative Western blots and summary data for sarcoplasmic/endoplasmic reticulum Ca^2+^ ATPase 2a, CaMKII phosphorylated at Thr286, RyR2, total phospholamban, RYR phosphorylated at Ser2808, phospholamban phosphorylated at Thr17, protein kinase A, Cav1.2, NLRP3, nuclear factor-κB, and interleukin-1β expression in the RVOT tissues of the control (healthy rats; *n* = 7), experimental (rats with myocarditis; *n* = 7), and treatment (MCC950-treated rats with myocarditis; *n* = 7) groups. Glyceraldehyde-3-phosphate dehydrogenase served as a loading control. CaMKII, Ca2 + /calmodulin-dependent protein kinase II; RYR, ryanodine receptor; RVOT, right ventricle outflow tract

We investigated Ca^2+^ regulatory proteins that mediate the detrimental effects of myocarditis on Ca^2+^ homeostasis. The expression levels of NLRP3, NF-κB, IL-1B, RyR2, RyR2 phosphorylated at serine-2808 (pRyR2-S2808), and pCaMKII were higher but that of SERCA2a was lower in the RVOTs of the experimental group than in the RVOTs of the control group (Fig. 6). However, the expression levels of protein kinase A, phospholamban, Cav1.2, and phospholamban phosphorylated at T17 were similar in these two groups. After MCC950 treatment, we discovered upregulated expression of IL-1B, RyR2, pRyR2-S2808, and pCaMKII in the rat RVOTs (Fig. 6). Treatment of the experimental rats with anti-NLRP3 antibodies blocked the myocarditis-induced activation of Ca^2+^ signaling, implicating NLRP3 in the dysregulation of Ca^2+^ signaling in myocarditis. These findings indicate that myocarditis disrupts Ca^2+^ homeostasis in the RVOT by upregulating the expression of inflammatory proteins and Ca^2+^-handling proteins—such as NLRP3, RyR2, pRyR2-S2808, and pCaMKII—and by downregulating the expression of SERCA2a. Because these dysregulations were mitigated in the treatment group, we believe that MCC950 can be used to target NLRP3 to mitigate myocarditis-induced abnormalities in Ca^2+^ signaling.

## Discussion

VT is a major cause of sudden cardiac death and mortality, both in the general population and patients with myocarditis [28, 29]. Nonsustained VT and frequent PVCs are associated with an increased risk of cardiovascular mortality [30]. Pathological data from the present study revealed that cardiac regions with higher levels of NLRP3 inflammasome activity are more susceptible to VA. This suggests a direct correlation between NLRP3 activity and arrhythmogenic events in rats with myocarditis. Our in vitro experiments indicated higher incidence of PVCs and VT in the experimental group than in the control group. In addition, we found that the RVOT is more susceptible than are other ventricular regions to myocarditis-induced arrhythmogenesis. This observation suggests that autoimmunity-induced myocarditis leads to arrhythmia in the RVOT, likely due to inflammation and electrolyte imbalance, particularly that involving abnormal Ca^2+^ handling. Notably, embryological studies have unraveled different origins of RVOT, RVA, and LV cardiomyocytes [31, 32]. The right ventricle, particularly the RVOT, may be more dependent than the LV on dynamic phosphorylation and dephosphorylation activities [33]. Our experimental group exhibited higher levels of NLRP3 expression and inflammation in the right ventricle than in the LV, and these higher levels potentially contributed to the increased susceptibility of the RVOT to myocarditis-associated arrhythmia. These findings elucidate the mechanisms underlying arrhythmogenesis in rats with myocarditis, highlighting the role of inflammation and NLRP3 activation, particularly in the RVOT. An improved understanding of these mechanisms may guide future therapeutic strategies for myocarditis-associated arrhythmia.

In patients with myocarditis, VA can occur at various stages of inflammation. During the acute phase, inflammation mediates the development of arrhythmogenic foci throughout the myocardium. Tissue edema increases the extracellular space, reducing the local myocardial conduction velocity in certain regions and thereby perhaps facilitating the formation of re-entry circuits [34, 35]. Abnormal cycling of Ca^2+^, downregulation of potassium channels, altered expression of gap junction proteins, and disruption of dystrophin increase the risk of VT by prolonging the duration of AP and creating arrhythmogenic conditions [34, 36]. In patients with late-onset VA after myocarditis, the regular and monomorphic type of VA is more consistent with stable scar-related re-entry circuits than are other types [8, 37]. The myocarditis-induced disruption of Ca^2+^ regulation in cardiac cells, which is exacerbated by caffeine exposure, can further predispose patients to arrhythmia [38]. In the present study, these effects were observed only in the experimental group, suggesting that the arrhythmic potential of caffeine is amplified by the combined effects of sympathetic activation and Ca^2+^ imbalance in rats with myocarditis. In myocarditis, NLRP3 expression increases as part of the immune response; we observed higher NLRP3 levels in the experimental and treatment groups than in the control group. MCC950 blocks the NLRP3 activator–induced release of IL‑1β [39–41] and inhibits the NLRP3 inflammasome by directly targeting the NATCH domain of NLRP3, thereby interfering with the function of the Walker B motif and preventing the conformational change and oligomerization of NLRP3 [42, 43]**.** Thus, MCC950 inhibits the activity, but not the expression, of the NLRP3 inflammasome [43]. Central to this process is NLRP3, which, upon activation, forms the inflammasome complex, leading to the release of proinflammatory cytokines such as IL-1β and IL-18. These cytokines play key roles in promoting the activation and proliferation of T cells, thus exacerbating the autoimmune response against cardiac myosin, resulting in myocardial inflammation and damage typical of myocarditis. In this study, we targeted NLRP3 signaling to prevent the activation of this inflammatory cascade and block the production of proinflammatory cytokines. Western blotting revealed elevated IL-1β levels in the experimental group, but the NLRP3 inhibitor MCC950 largely prevented the elevation of these levels. This finding underscores the potential of NLRP3 inhibition in managing myocarditis-induced inflammatory responses. We further found a more pronounced inflammatory response in the RVOT than in the LV, which is partly attributable to the higher contents of epicardial fat tissue in the RVOT [44, 45]. Adipose tissue in the heart mediates immune and inflammatory responses by secreting various cytokines and chemokines that amplify local inflammatory reactions [46, 47]. The RVOT typically has high content of adipose tissue, which is more prone to inflammation than are other types of tissue. The high content of adipose tissue in the RVOT may serve as a focal point for the infiltration of inflammatory cells and the production of cytokines in myocarditis. Thus, RVOT adipose tissue may be an immunologically active site and may have contributed to the regional heterogeneity observed in our study. In the experimental group, the triggered activity levels and VT incidence were significantly higher in the RVOTs than in did the RVAs and LVs. This finding suggests that in patients with acute myocarditis, VA is more likely to originate from the RVOT than from any other cardiac region. The incidence of VT was higher in the RVAs of the experimental group than in the RVAs of the control group; however, the difference was nonsignificant. This finding indicates the emergence of a persistent substrate for the formation of re-entry circuits, likely through replacement-type fibrosis and scar formation. However, myocarditis exerted a less pronounced effect on LV arrhythmogenesis, perhaps because of the large thickness of the LV myocardium. Compared with the RVOT, the LV has a thicker myocardium with a lower level of adipocyte infiltration, which buffers against the infiltration of inflammatory cells. These features of the LV contribute to its resistance to inflammation-induced arrhythmogenesis and enable it to withstand and recover from inflammation-induced stress and damage [48]. Rapamycin can induce a Ca^2+^ leak, thus increasing the risk of cardiac arrhythmia. This effect is attributable to rapamycin-mediated inhibition of the mechanistic target of rapamycin pathway, which is essential for maintaining the Ca^2+^ balance in cardiac myocytes. By disrupting the intracellular regulation of Ca^2+^, rapamycin triggers Ca^2+^ leakage and arrhythmogenesis [49, 50]. Our findings indicate variation in arrhythmogenicity across the cardiac regions of rats with myocarditis. The RVOT appears to be particularly susceptible to VA, whereas the RVA and LV exhibit varying degrees of susceptibility. Understanding the regional differences and the underlying pathophysiological mechanisms can facilitate the diagnosis and management of arrhythmia in patients with myocarditis.

COVID-19 can directly infect cardiomyocytes by attaching to angiotensin-converting enzyme 2 receptors, which are abundant in the heart, rendering the heart vulnerable to viral invasion [51]. Once inside the cells, the SARS-CoV-2 virus replicates and causes inflammation and damage to the cardiac muscle, potentially leading to myocarditis. In addition, the release of proinflammatory cytokines in response to COVID-19 results in widespread inflammation across organs, including the heart; this explains the association between COVID-19 and myocarditis [52]. COVID-19-associated myocarditis is a consequence of the virus’ presence and activity, mediated through inflammatory and immune responses, rather than of an autoimmune response, such as that observed in our animal model. Similar to COVID-19-associated myocarditis, autoimmunity-associated myocarditis results in upregulated activation of the NLRP3 and NF-κB pathways in addition to an increase in IL-1β level. This suggests common inflammatory mechanisms between COVID-19-associated and autoimmunity-associated myocarditis [51, 52]. Activation of the NLRP3 inflammasome drives the inflammatory response in both scenarios, leading to the secretion of cytokines. These findings indicate inflammation dynamics that are likely applicable to the cardiac implications of COVID-19. Patients with myocarditis commonly have prolonged QRS duration and QTc interval, which are attributable to an increase in the ventricular APD. This prolongation has been shown to render these patients susceptible to arrhythmogenesis [10, 53]. Prolonged QRS duration and QTc interval are associated with higher risks of fulminant disease and in-hospital mortality [53]. These findings corroborate ours: the QRS duration and QTc interval were longer in the experimental group than in the control and treatment groups. Furthermore, the APD was longer in the RVOTs of the experimental group than in the RVOTs of the control and treatment groups. A prolonged APD may increase the risk of VA in the RVOT, particularly in patients with long QT syndrome [54, 55]. Prolongation of APD further exacerbates the reductions in diastolic filling and stroke volume at high pulse rates [56]. A prolonged QT interval may increase the risk of VA by enhancing triggered activity, particularly through early depolarization [57]. Our findings revealed a more pronounced inflammatory response in the RVOT than in the RVA and LV, which may explain the observed differences in APD morphology. The RVOT may be more vulnerable to inflammatory stress because of its distinct histopathological characteristics, including its adipose tissue distribution. This finding is supported by that of a study indicating that inflammation can extend APD_90_ by modifying essential ion channels and Ca^2+^ handling [58]. The MCC950-mediated reduction in VA incidence in the RVOTs of the experimental group suggests that inhibiting NLRP3 with MCC950 reduces the risk of arrhythmia by suppressing arrhythmogenesis in the RVOT.

Our results indicated significantly larger SR Ca^2+^ leaks from the RVOTs of the experimental group than those of the control group. A larger Ca^2+^ leak may be associated with smaller [Ca^2+^]_i_ transients and lower SR Ca^2+^ levels. The role of CaMKII as an intracellular Ca^2+^ sensor is particularly noteworthy in this context. CaMKII functions as a key regulator of immune and inflammatory responses, which mediate the alternations in Ca^2+^ handling [59, 60]. An increase in Ca^2+^ leakage is a key mechanism that contributes to the increased risk of arrhythmia in the RVOT, which emphasizes the role of Ca^2+^ dynamics in myocarditis-induced arrhythmogenesis [45]. In our study, the RVOT expression levels of RyR2, pRyR2 s2814, and pCaMKII were higher and that of SERCA2a was lower in the experimental group than in the control group. The RVOTs of the experimental group also had reduced SR Ca^2+^ levels and relatively large NCX current, which suggests a strong correlation between NCX current and Ca^2+^ homeostasis [61]. SERCA2a—which is modulated by phospholamban, sarcolipin, and CaMKII-mediated direct phosphorylation—facilitates the storage of Ca^2+^ within the SR [62]. Thus, the reuptake of cytosolic Ca^2+^ into the SR can be reduced by suppressing SERCA2a function. Hyperactive RyR–mediated leakage of Ca^2+^ from the SR may contribute to the incidence of VA [63]. Ca^2+^ overload can lead to inactivation of *I*_Ca-L_ in the RVOT, thereby inhibiting the influx of Ca^2+^ into cardiomyocytes [64]. In addition to the level of SERCA2a, that of Ca^2+^ in the SR is reduced due to the depletion of Ca^2+^ stores; eventually, a reduction is noted in [Ca^2+^]_i_ transients because of the downregulation of the RYR2-mediated release of Ca^2+^ from the SR. In our study, the upregulated expression of pCaMKII in the RVOTs of the experimental group may have increased the leakage of Ca^2+^. Nonetheless, MCC95 mitigated the myocarditis-induced leakage of Ca^2+^; this finding suggests that activation of CaMKII is crucial for the effects of myocarditis on RVOT cardiomyocytes.

CaMKII substantially enhances *I*_Na-Late_ [65], thereby increasing intracellular Na^+^ loading and promoting arrhythmogenesis. Increased *I*_Na-Late_ has been implicated in the pathophysiology of acquired cardiac diseases, such as myocardial ischemia [66] and heart failure [67]. The present study revealed a significantly larger *I*_Na-Late_ in the RVOTs of the experimental group than in those of the control group. An increase in *I*_Na-Late_ may disrupt the balance between Na^+^ efflux and Ca^2+^ influx, resulting in intracellular Ca^2+^ overload [68]. However, in our study, MCC95-treated rats with myocarditis had smaller *I*_Na-Late_ than did their untreated counterparts. These findings suggest that MCC950 partially mitigates the risk of VA incidence in the RVOT by reducing *I*_Na-Late_ and thus highlight the roles of *I*_Na-Late_ and its MCC950-mediated modulation in the pathophysiology of myocarditis-associated arrhythmia. By targeting *I*_Na-Late_ and its downstream effects on intracellular Ca^2+^ handling, MCC950 may prevent arrhythmogenesis in the RVOTs of patients with myocarditis.

**Fig. 7:**
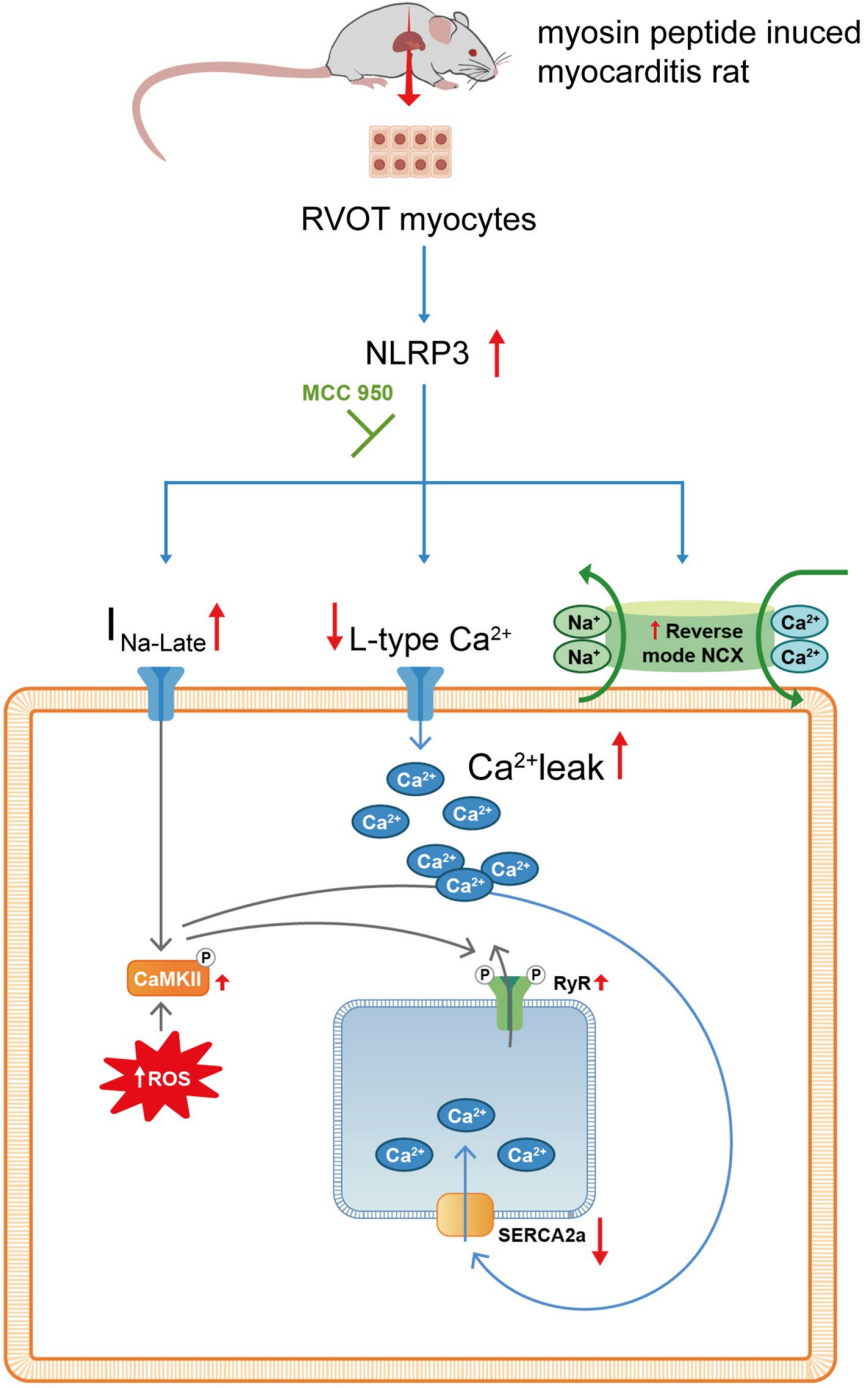
Potential mechanisms underlying the role of NLRP3 signaling in myocarditis. MCC950 treatment may reverse myocarditis-induced Na^+^–Ca^2+^ dysregulation by mitigating the alterations in levels of ROS, CMKII, RyR2, and ionic channels in cardiomyocytes. *I*_Na-Late_: late Na^+^ current; NCX, Na^+^–Ca^2+^ exchanger; ROS, reactive oxygen species; RyR2, ryanodine receptor 2; RyR2-pS2808, RyR2 phosphorylated at serine 2808; SR, sarcoplasmic reticulum; SERCA, sarcoplasmic reticulum ATPase

Activation of the NLRP3 inflammasome induces CaMKII signaling, which promotes cardiac remodeling [69]. Consistent with this finding, we observed significantly higher levels of NLRP3 inflammasome activation and CaMKII expression in the experimental group than in the control group. Inflammation-induced oxidative stress is a key mediator of myocarditis progression [70]. In our study, MCC950 downregulated the expression of ROS and CaMKII and the rate of arrhythmogenesis in the RVOTs of rats with myocarditis. These findings suggest that the activation of NLRP3-induced CaMKII signaling and the associated increase in ROS levels may contribute to the development of VA in rats with myocarditis. However, MCC950 effectively suppressed the activity of NLRP3, ROS, and CaMKII, thereby restoring NCX, *I*_Na-Late_, and Ca^2+^ levels in the RVOTs of the experimental group (Fig. 7).

### Limitations

The present study has some limitations. First, this study primarily relied on a rat model of myosin peptide–induced myocarditis. Although this model provides valuable insights, myocarditis response may differ between rats and humans and vary depending on etiology. Therefore, our findings should be interpreted with caution when extrapolating to human myocarditis. Second, we administered MCC950 for a fixed duration (14 days). Further investigations into the optimal treatment duration and long-term effects of MCC950 are needed to enhance the clinical relevance of our findings. Third, this study primarily focused on NLRP3 signaling; however, myocarditis is a multifaceted condition influenced by various factors, such as viral infection, autoimmune response, and genetic predisposition. The contribution of these factors to RVOT arrhythmogenesis should not be overlooked. Fourth, our study mainly clarified the mechanisms underlying RVOT arrhythmogenesis. Future studies should investigate the clinical correlations of our findings with those observed in human patients with myocarditis. Finally, we studied arrhythmogenesis primarily in the RVOT, not the left ventricular outflow tract. This limitation necessitates further investigation.

## Conclusion

Our findings suggest that RVOT cardiomyocytes are predisposed to myocarditis and associated arrhythmogenesis. Inhibition of NLRP3 may attenuate myocarditis-induced dysregulations in Ca^2+^ and Na^+^ levels by downregulating the expression of ROS and CaMKII, thereby normalizing key electrical and structural parameters. Therefore, in patients with myocarditis, NLRP3 signaling may be targeted to reduce the risk of VA.

## Data Availability

Not applicable.

## Abbreviations

AP: Action potential
APA: Action potential amplitude
APD: Action potential duration
Ca^2+^: Calcium
CaMKII: Ca^2+^/calmodulin-dependent protein kinase II
ECG: Electrocardiography
*I*_Na-Late_: Late sodium
IL: Interleukin
LV: Left ventricle
NCX: Na^+^–Ca^2+^ exchanger
NLRP3: Nucleotide oligomerization domain-like receptor family protein 3
PVC: Premature ventricular contraction
RVA: Right ventricular apex
RVOT: Right ventricular outflow tract
ROS: Reactive oxygen species
RyR2: Ryanodine receptor 2
RyR2-pS2808: RyR2 phosphorylated at serine 2808
SR: Sarcoplasmic reticulum
SERCA: Sarcoplasmic reticulum ATPase
VA: Ventricular arrhythmia
VT: Ventricular tachycardia
